# Whole-Genome Analysis Reveals That the Nucleoid Protein IHF Predominantly Binds to the Replication Origin *oriC* Specifically at the Time of Initiation

**DOI:** 10.3389/fmicb.2021.697712

**Published:** 2021-08-12

**Authors:** Kazutoshi Kasho, Taku Oshima, Onuma Chumsakul, Kensuke Nakamura, Kazuki Fukamachi, Tsutomu Katayama

**Affiliations:** ^1^Department of Molecular Biology, Graduate School of Pharmaceutical Sciences, Kyushu University, Fukuoka, Japan; ^2^Department of Biotechnology, Toyama Prefectural University, Toyama, Japan; ^3^Graduate School of Biological Sciences, Nara Institute of Science and Technology, Nara, Japan; ^4^Department of Life Science and Informatics, Maebashi Institute of Technology, Maebashi, Japan

**Keywords:** *Escherichia coli*, cell cycle, IHF, GeF-seq, *oriC*

## Abstract

The structure and function of bacterial chromosomes are dynamically regulated by a wide variety of nucleoid-associated proteins (NAPs) and DNA superstructures, such as DNA supercoiling. In *Escherichia coli*, integration host factor (IHF), a NAP, binds to specific transcription promoters and regulatory DNA elements of DNA replication such as the replication origin *oriC*: binding to these elements depends on the cell cycle but underlying mechanisms are unknown. In this study, we combined GeF-seq (genome footprinting with high-throughput sequencing) with synchronization of the *E. coli* cell cycle to determine the genome-wide, cell cycle-dependent binding of IHF with base-pair resolution. The GeF-seq results in this study were qualified enough to analyze genomic IHF binding sites (e.g., *oriC* and the transcriptional promoters of *ilvG* and *osmY*) except some of the known sites. Unexpectedly, we found that before replication initiation, *oriC* was a predominant site for stable IHF binding, whereas all other loci exhibited reduced IHF binding. To reveal the specific mechanism of stable *oriC–*IHF binding, we inserted a truncated *oriC* sequence in the *terC* (replication terminus) locus of the genome. Before replication initiation, stable IHF binding was detected even at this additional *oriC* site, dependent on the specific DnaA-binding sequence DnaA box R1 within the site. DnaA oligomers formed on *oriC* might protect the *oriC*–IHF complex from IHF dissociation. After replication initiation, IHF rapidly dissociated from *oriC*, and IHF binding to other sites was sustained or stimulated. In addition, we identified a novel locus associated with cell cycle-dependent IHF binding. These findings provide mechanistic insight into IHF binding and dissociation in the genome.

## Introduction

Within bacterial cells, chromosomal DNA forms a dynamic and highly condensed structure called the nucleoid. In *Escherichia coli*, the nucleoid is organized by a wide variety of nucleoid-associated proteins (NAPs), RNA, and DNA supercoiling. Major bacterial NAPs such as integration host factor (IHF), heat unstable (HU), and factor for inversion stimulation (Fis), along with DNA supercoiling, regulate various cellular events such as DNA replication, transcription, recombination, and nucleoid condensation (Dillon and Dorman, 2010; Seah et al., 2014). HU, one of the most abundant NAPs, binds to AT-rich DNA without sequence specificity (Ali Azam et al., 1999; Dillon and Dorman, 2010). IHF, a hetero-dimeric protein that consists of α and β subunits (encoded by *ihfA* and *ihfB*, respectively), binds to DNA in a sequence-specific manner and causes sharp (>120°) bending (Rice et al., 1996; Aeling et al., 2006). Fis, which is expressed specifically in log-phase cells (Ali Azam et al., 1999; Dillon and Dorman, 2010), also binds DNA in a sequence-specific manner, but bends DNA more moderately (∼60°) (Stella et al., 2010). The chromosomal DNA is not randomly condensed but instead dynamically forms specific structures during DNA replication and segregation (Toro and Shapiro, 2010); however, the regulatory mechanism underlying these structural changes remains unclear.

**FIGURE 1:**
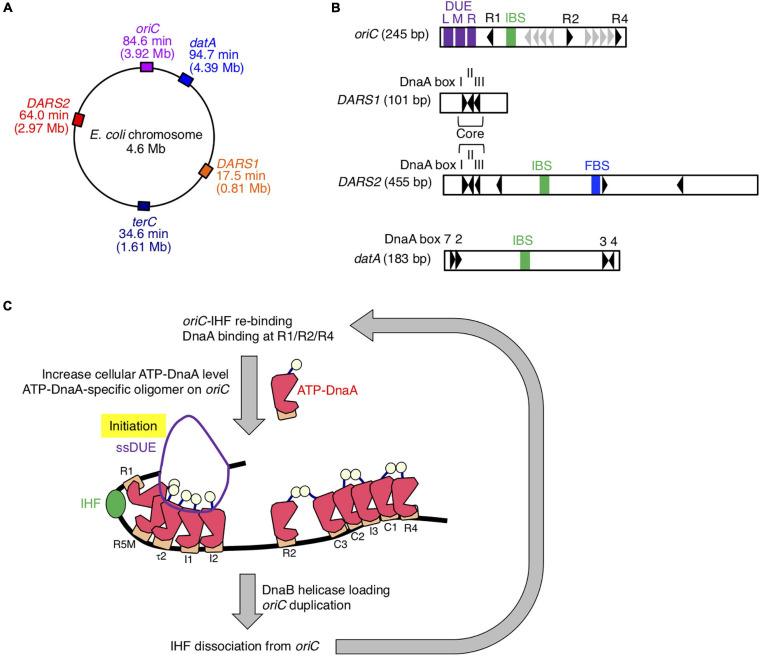
Role of IHF binding at *oriC*. **(A)** The locations of *oriC*, *datA*, *DARS1*/*2*, and *TerC* loci are indicated on the chromosome. **(B)** Schematic representation of the structures of *oriC*, *datA*, and *DARS1*/*2* are described. Black arrowheads represent DnaA-binding sites (DnaA boxes) that match the 9-mer consensus sequence completely or with only a single mismatch. Gray arrowheads in *oriC* indicate low-affinity ATP–DnaA-binding sites R5, I1–3, τ2, and C1–3. For simplicity, a non-essential τ1 site partially overlapping with IHF-interacting region including IHF-binding sequence (IBS; green bars) is omitted (Sakiyama et al., 2017). DnaA boxes 2, 3, 4, and 7 in *datA* and “Core” DnaA boxes I–III in *DARS1*/*2* are essential for their activities. IBS, Fis-binding sequence (FBS; blue bar), and AT-rich repeats L, M, and R (DUE; purple bars) are also indicated. **(C)** Role and timing of IHF binding at *oriC*. IHF binds to a unique IBS and bends DNA during the pre-initiation period. At the initiation stage, ATP–DnaA becomes more abundant and forms specific oligomers on *oriC*. The left DnaA pentamer with bound IHF unwinds the DUE to initiate replication. After replication initiation, IHF quickly dissociates from *oriC*. DnaA is shown as pink polygon (domain III) with light yellow ball (domain I) and orange rectangle (domain IV). IHF is also shown as a green ball.

Initiation of chromosomal DNA replication is rigidly controlled to ensure that it occurs only once during the cell cycle (Katayama et al., 2010, 2017; Skarstad and Katayama, 2013; Riber et al., 2016). The initiator protein DnaA and IHF play crucial roles in initiating replication at the chromosomal origin, *oriC* (Figures 1A–C). DnaA forms complexes with ATP or ADP, and ATP–DnaA is active in initiation (Shimizu et al., 2016; Sakiyama et al., 2017). *oriC* contains an AT-rich duplex unwinding element (DUE), a single IHF-binding sequence (IBS), and a DnaA oligomerization region (DOR) that contains two subregions with oppositely oriented clusters of DnaA-binding sites (DnaA boxes; Figure 1B; Ozaki et al., 2012; Noguchi et al., 2015). The high-affinity DnaA boxes R1 and R4 at the outer edges of the DOR are oriented in opposite directions, and cooperative binding of ATP–DnaA molecules to lower affinity sites in the Left and Right DORs results in the formation of two DnaA subcomplexes (Figure 1C). IHF specifically binds to the 13-mer IBS and bends DNA sharply (Shimizu et al., 2016; Sakiyama et al., 2017). At initiation, binding of IHF and ATP–DnaA molecules to *oriC* induces a conformational change and local unwinding of the *oriC* DUE (Figure 1C), followed by loading of the DNA replication machinery.

The level of ATP–DnaA in the cell is tightly regulated, peaking at the stage of replication initiation (Kurokawa et al., 1999). During replication, DnaA-bound ATP is hydrolyzed by a complex containing Hda and the DNA-loaded clamp subunit of DNA polymerase III holoenzyme, yielding initiation-inactive ADP–DnaA (Kato and Katayama, 2001; Katayama et al., 2010). This replication-coupled negative feedback system is termed regulatory inactivation of DnaA (RIDA). In addition, the specific chromosomal locus *datA*, which contains four DnaA boxes and an IBS, is required to prevent untimely initiations (Figures 1A,B; Nozaki et al., 2009). Recently, we showed that ATP–DnaA molecules form specific complexes on IHF-bound *datA*, which stimulates hydrolysis of DnaA-bound ATP (Kasho and Katayama, 2013; Kasho et al., 2017). *datA*–IHF binding specifically occurs at the post-initiation stage of the cell cycle. This system for timely inactivation of DnaA, termed DDAH (*datA*-dependent DnaA–ATP hydrolysis), plays a supplemental role to RIDA in timely yielding of ADP–DnaA.

In contrast to *datA*, the DnaA-binding chromosomal loci called *DARS*s (DnaA-reactivating sequences) increase the level of ATP–DnaA by promoting nucleotide exchange of ADP–DnaA (Figures 1A,B; Fujimitsu et al., 2009). The *E. coli* chromosome contains at least two *DARS*s, *DARS1*, and *DARS2*, which are required for timely initiation of replication during the cell cycle. *DARS1* and *DARS2* share three highly conserved “Core” DnaA boxes that are necessary for nucleotide exchange (Fujimitsu et al., 2009; Sugiyama et al., 2019). In contrast to *DARS1*, *DARS2* requires two activator proteins, IHF and Fis (Kasho et al., 2014). *DARS2*–IHF binding is temporally regulated to occur at the pre-initiation stage of the cell cycle. Thus, IHF bindings to *oriC*, *datA*, and *DARS2* are regulated such that they occur at different times during the cell cycle; however, the regulatory mechanism and the genome-wide dynamics of timely IHF binding during the cell cycle remain to be elucidated.

In this study, we utilized GeF-seq, a unique method for identifying protein-binding sites with base-pair resolution (Chumsakul et al., 2013), to identify genome-wide distribution of IHF-binding sites. Based on results obtained by combining GeF-qPCR, GeF-seq and cell cycle synchronization, we identified novel cell cycle-coordinated IHF dynamics: at the replication initiation stage, IHF specifically binds to *oriC* and dissociates from other genomic IHF-binding loci, whereas after replication initiation, IHF is dissociated from *oriC*, and many IHF molecules stably bind to other binding loci. We analyzed IHF-binding consensus sequences at each cell cycle stage and suggest that IHF can temporarily bind to a secondary IBS on *oriC* at the stage of replication initiation. Further mechanistic analysis of *oriC* revealed that the presence of DnaA box R1, but not the chromosomal location of *oriC*, was required for stable *oriC*–IHF binding at the initiation stage. In addition, we comprehensively analyzed genomic IBS and found novel binding loci in the *ttcA* gene that are likely cell cycle-specific. Based on these findings, we propose a model of the specific mechanism involved in stable IHF binding at *oriC* and hypothesize that the modes of genomic IHF binding drastically change during the cell cycle, potentially having a global effect on the dynamics of nucleoid structures and functions.

## Materials and Methods

### Bacterial Strains and Cultures

For GeF-seq, *E. coli* SH022 (*dnaC2 ihfA-cHis12*) cells were used (Table 1; Kasho et al., 2014; Inoue et al., 2016). To introduce *oriC*ΔDUE at the *terC* locus, a pBR322-based plasmid with *oriC*ΔDUE and *kan* (kanamycin resistant) gene flanking *frt* sequences (pKX136) was constructed. The DNA fragment bearing the *oriC*ΔDUE-*frt-kan* with the chromosomal sequence of the *terC* -proximal locus (intergenic region between *pntA* and *ydgH*; Ter-2 locus in Inoue et al., 2016) was amplified using primers TERori1-U and TERori-L (Table 2). Site-directed recombination was performed using SH022 cells, and the *frt-kan* region was removed using pCP20 to yield KX237 (*dnaC2 ihfA-cHis12* TER-*oriC*ΔDUE) and KX238 [*dnaC2 ihfA-cHis12* TER-*oriC*Δ(DUE-R1)] (Table 1; Datsenko and Wanner, 2000). To introduce *oriC*Δ(DUE-R1) at the *terC* locus, *oriC*Δ(DUE-R1)*-frt-kan* in pKX136 was amplified using primers TERori2-U and TERori-L, and similarly inserted into the SH022 genome.

**TABLE 1:** list of *E. coli* strains.

**Strains**	**Genotypes**	**References**
SH022	MG1655 *ihfA-cHis12 dnaC2 zjj18*::*cat*	Kasho et al., 2014
KYA018	MG1655 *dnaC2 zjj18*::*cat*	Kasho and Katayama, 2013
KX237	SH022 *zdg7*::*oriC*ΔDUE	This study
KX238	SH022 *zdg7*::*oriC*Δ(DUE-R1)	This study

**TABLE 2:** List of oligonucleotides.

**Names**	**Sequences**
ORI_1	CTGTGAATGATCGGTGATC
KWoriCRev	GTGGATAACTCTGTCAGGAAGCTTG
RTYLCC-L	GGCGTGGTAAAGGGTATCG
RTYLCC-R	TCTGCGGGGTGATGGTAAAG
ilvG-U	TCCTCGGTTATGTTTTTAAGGTC
ilvG-L	TGCACTTGGACGAGGAAAG
rhlB-U	TACGTCACGACCCGCCAG
rhlB-L	CATCCGAAGGTTGTAGAAGC
glnH-U	AATGGTGCATCTTCAGGGTATTG
glnH-L	CACATATATGAAAAAATCGTGCCAG
osmY-U	ATCACAATTTTGAAACCGCTC
osmY-L	CTGTCAATTTCCCTTCCTTATTAGC
TERori1-U	GATAAAGACTGATAATTGTCTTCGACGGTCGGGT AAAACGAGACAATCGCACTGCCCTGTGGATAAC
TERori2-U	GATAAAGACTGATAATTGTCTTCGACGGTCGGGT AAAACGAGACACAAGGATCCGGCTTTTAAGATCAAC
TERori-L	TGTATAAGTTAATTTAATGTTAAGTAGTGATTCGTG CCGGGGCGACCATATGAATATCCTCCTTAGTTCC
RT-TERoriC-U	CTCGCAAAATATTAACGATTCAGCCG
RT-TERoriC-L	TGTCTCGTTTTACCCGACCG
RT-NoriC-U2	GATCTGTTCTATTGTGATCTCTTATTAGGATCG
RT-NoriC-L2	CACAGTTAATGATCCTTTCCAGGTTG

Cell cultivation and cell cycle synchronization were performed according to a previously described method with minor modifications (Kasho and Katayama, 2013; Kasho et al., 2014; Inoue et al., 2016). To synchronize the *E. coli* cell cycle, cells were grown in supplemented M9 medium at 30°C, the permissive temperature for *dnaC2*, until the A_660_ of the culture reached 0.03, followed by further incubation at 38°C, the restrictive temperature, for 90 min. Cells were immediately cooled to 30°C by addition of ice-cold medium and then incubated for an additional 10 or 20 min. Cell samples were withdrawn at the indicated time points, collected, and crosslinked with 3% (final) formaldehyde for 5 min.

### *In situ* DNase I Digestion, His-Tag Affinity Purification of IHF–DNA Complexes, and Sequencing

To hydrolyze the cell wall without osmotic burst, cells were treated with 1 mg/ml egg white lysozyme in 2 ml isotonic PeriPrep buffer [200 mM Tris–HCl (pH 8.0), 50%(v/v) sucrose] in the presence of 1 mM phenylmethylsulfonyl fluoride (PMSF). After incubation for 15 min at 37°C with mixing, cells were collected by centrifugation at 7,000 rpm for 5 min at 4°C, and then resuspended in 550 μl king2 buffer [100 mM Tris–HCl (pH 7.5), 200 mM NaCl, 1% (v/v) Triton X-100, 0.1% (w/v) Na-deoxycholate, 0.2% (w/v) Brij 58, and 20% (v/v) glycerol]. *In situ* DNase I treatment was performed by adding 50 μl MgCa buffer (100 mM MgCl_2_ and 50 mM CaCl_2_), 100 μg RNase A, and 20 units of DNase I (New England Biolabs, Ipswich, Massachusetts, United States) and incubating at 37°C for 15 min. Reactions were stopped by adding 3 ml UT buffer [50 mM HEPES-KOH (pH 7.6), 250 mM NaCl, 0.5% (v/v) Triton X-100, 5 mM imidazole, 5 mM β-mercaptoethanol, 9 M urea, and 1 mM PMSF]. The resultant suspensions were sonicated for 2 min (4 s “on”/10 s “off,” 30 times, output 2), and cell debris was removed by centrifugation at 12,000 rpm for 5 min at 4°C. A portion (200 μl) of the resultant supernatant was used to check DNA size by 2% agarose gel electrophoresis. The rest (3.5 ml) was mixed with 100 μl Dynabeads His-tag Isolation and Pulldown (Life Technologies, Carlsbad, California, United States), followed by incubation at 4°C overnight with a gentle rotation. Beads and bound materials were washed seven times with UT buffer, resuspended in 200 μl elution buffer [100 mM Tris–HCl (pH 7.5), 500 mM imidazole, 1% (w/v) SDS, and 10 mM dithiothreitol]. Proteins were degraded by Proteinase K treatment at 42°C for 2 h, followed by further incubation at 65°C for 6 h for de-crosslinking. After removal of proteins by phenol–chloroform–isoamyl alcohol extraction, DNA was recovered by ethanol precipitation in the presence of glycogen and resuspended in 10 μl nuclease-free water.

The DNA library for next-generation sequencing (NGS) was produced using the NEB Next DNA Sample Prep Reagent kit (New England Biolabs) according to the manufacturer’s instructions for “Preparing Samples for Sequencing Genomic DNA” (Illumina). The DNA fragments were then purified using a WIZARD SV Gel and PCR Clean-Up System (Promega), and amplified by 15 cycles of PCR. The sequence of the library was then determined by BioAnalyzer (Agilent Technologies).

Short-read sequencing was performed by BGI (Shenzhen, Guangdong, China) with the paired-end procedure (100 bp × 2) on an Illumina HiSeq 2000 instrument (Illumina, San Diego, California, United States). The fastq files of forward and reverse short-read sequencing for each DNA library were concatenated for read mapping, and IHF-binding regions were detected using the pmapsr program (see following section).

### Determination of Highest IHF-Binding Regions and Determination of IHF-Binding Motifs

The regions protected by IHF in the *E. coli* genome, which would be sandwiched between the edges of DNase I digestion corresponding to the 5′ and 3′ ends of the forward and reverse short reads, were identified precisely using the pmapsr program (Chumsakul et al., 2013). The DNase I-digested short fragments were estimated to be 70–110 bp long; because we performed 100 bp Illumina sequencing, the reads frequently included the primer sequences added for DNA library construction (see before section), causing severe mismatches for mapping of the reads to the *E. coli* K-12 MG1655 genome (reference sequence) and decreasing the number of mapped reads. To reduce the number of unmapped reads, we initially mapped reads using the mpsmap program permitting 35 bp mismatch in order to be able to map the reads, including the primer sequences (Chumsakul et al., 2013). We then determined the boundaries of the primer sequences and the homologous sequences to the reference genome in the forward and reverse short reads, which represent the 5′ or 3′ ends of DNase I-digested short fragments. We identified the 70–110 bp regions sandwiched between the 5′ or 3′ ends with high read depths as candidate IHF-binding regions (see details described below).

Detection of highest IHF-binding regions was performed according to the instruction for the pmapsr program for GeF-seq analysis with minor modifications (the original version used in this study;^1^, the new version with some minor bug fix;^2^). We did not use the options -pbo, -pbt, or -pbs, which have been adapted to analyze randomly replicating cells and have a genome dosage bias from the replication origin to the termination site in exponentially growing cells. In this study, we used *dnaC2*-based cells to synchronize the replication cycle; consequently, such bias would not be present. We used the following options: -primer to detect primer sequence, -ewf 70 to set the minimal length of the binding regions to 70 bp, and -ewt 110 to set the maximal length of the binding regions to 110 bp; thus, we could detect the 5′ or 3′ ends of 70--110 bp regions protected from DNase I digestion, and -tp with appropriate values to set the threshold values, to select highest IHF-binding regions using the pmapsr program, which have the highest average read depths in IHF-binding regions. We set the threshold value at 10,000 reads for the 0 min dataset, and then adjusted the threshold values of other datasets to reflect the differences of mapped read numbers to the *E. coli* genome among datasets, i.e., 10,000 reads for the 0 min dataset (number of mapped reads, 15,289,849), 19,606 reads for the 10 min dataset (number of mapped reads, 29,977,846), 11,697 reads for the 20 min dataset (number of mapped reads, 17,885,459) and 12,784 reads for the Random dataset (number of mapped reads, 19,546,737). Those high threshold values allowed us to select comparable highest IHF-binding regions in different samples. Finally, we checked IHF-binding peaks and highest IHF-binding regions by visual inspection of IHF-binding profiles visualized on the Integrative Genomics Viewer (IGV;^3^) (Robinson et al., 2017). We removed probable artifacts of highest IHF-binding regions, i.e., contamination of *rRNA* operons including *rrnC* (Figure 3A), which occur frequently in chromatin immunoprecipitation (Waldminghaus and Skarstad, 2010).

**FIGURE 2:**
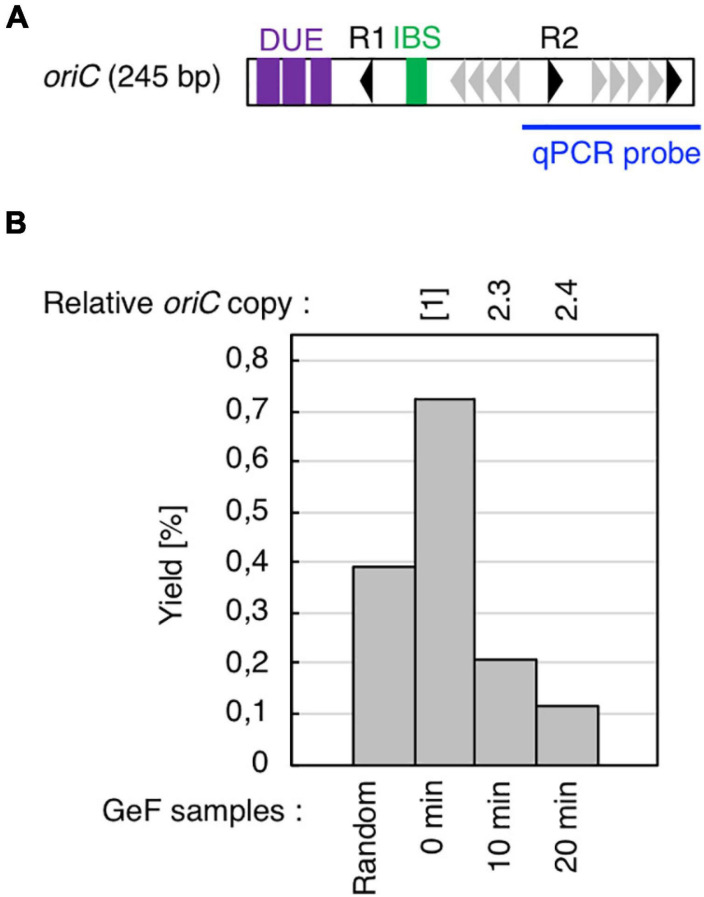
Cell cycle-specific IHF binding at *oriC* in GeF-qPCR. **(A)** Amplified region of *oriC* in qPCR experiments. Symbols used for *oriC* structure are shown as in Figure 1B. **(B)** IHF-GeF-qPCR of *oriC*. SH022 (*dnaC2 ihfA-cHis12*) cells growing at 30°C were transferred to 38°C and incubated for 90 min. The cells were then transferred to 30°C (Time 0) and further incubated for 10 or 20 min at 30°C. The relative *oriC* levels before and after Ni-affinity purification were determined using real-time qPCR, and yield was calculated (expressed as %). In addition, relative copies of the chromosomal *oriC* and *TerC* loci in the Input samples were quantified; the relative ratios of *oriC*/*ter* are expressed relative to the ratio at 0 min (determined as 1).

**FIGURE 3:**
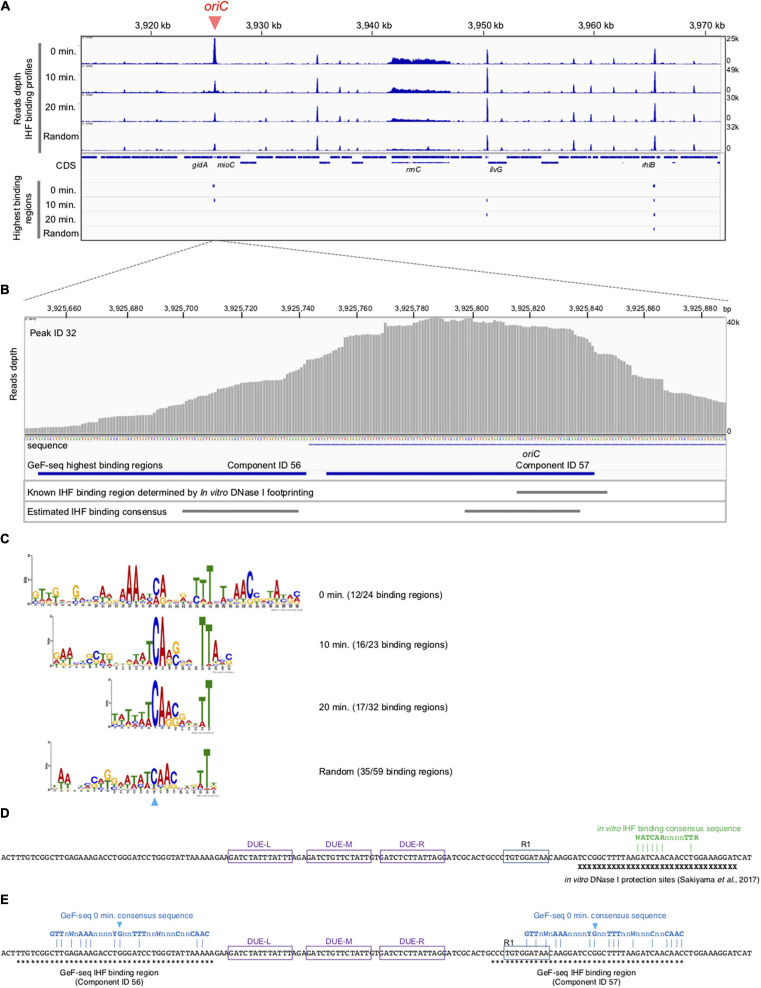
Cell cycle-specific IHF binding at *oriC* in GeF-seq. **(A)** The IHF-binding profile in the region including 3,920–3,970 kb part in the *E. coli* K-12 genome. Upper panels indicate the IHF-binding profiles in the 0 min, 10 min, 20 min, and Random samples. Read depth indicates the strength of IHF binding with the *E. coli* genome. To show each GeF-seq result, we altered the vertical scale in each dataset to allow direct comparison of peak height in each dataset shown in this figure. The vertical scale was expanded 1.96-fold (for the 10 min dataset), 1.2-fold (20 min), or 1.28-fold (Random) relative to the 0 min dataset, indicated at the right of the panels, because the mapped read number differed among samples: 15,289,849, 29,977,846, 17,885,459, and 19,546,737 in 0 min, 10 min, 20 min, and Random datasets, respectively. Therefore, this adjustment of the vertical scale makes it possible to directly compare the height of the IHF binding peaks visualized in figures without concerns about false differences due to differences in mapped read numbers. The threshold values are indicated as blue broken lines: 10,000, 19,606, 11,697, and 12,784 in 0 min, 10 min, 20 min, and Random datasets, respectively. Lower panels indicate highest IHF binding regions (Component IDs 56, 57, 58, and 59 in Supplementary Table 1). As shown here, the IBS in lower IHF-binding peaks has not been identified, although the lower-binding peaks may also have the IHF-binding consensus sequences and specifically interact with IHF. **(B)** Extension of Figure 3A at the *oriC* locus. Upper panel indicates read depth. At the bottom of this figure, the DNA sequences of the region and the location of the *oriC* locus are indicated. The lower panels indicate the locations of highest IHF-binding regions in the 0 min dataset (Components ID 56, 57 in Supplementary Table 1), the IBS determined by *in vitro* DNase I footprinting (Sakiyama et al., 2017), and the estimated IHF-binding consensus sequences based on the GeF-seq result for the 0 min dataset (highest IHF-binding regions). **(C)** Logos indicating consensus sequences estimated from highest IHF-binding regions in each dataset. On the right side of the Logos, datasets and the numbers of highest IHF-binding regions used to compute the Logos are indicated. The numbers indicate the proportion of binding regions including the consensus sequence (removing overlapping) among highest IHF-binding regions used for this analysis in each dataset (for instance, in the 0 min dataset, 12 highest IHF-binding regions include the consensus sequence indicated by the Logo, whereas a total of 24 highest-binding regions were detected. The sky-blue arrowhead indicates the position of the “C” residue at position 19 (0 min), which is highly conserved in all IBSs predicted in this study. **(D)** Known IBSs determined by *in vitro* DNase I footprinting. X indicates sequences in the region protected by IHF from DNase I digestion (Sakiyama et al., 2017). Three DUE elements (L, M, and R) and DnaA box R1 are indicated by purple or blue boxes. IHF binding consensus sequence previously determined by *in vitro* experiments is shown by green characters (Swinger and Rice, 2004). Dam-dependent methylation sequence GATC are labeled as yellow background. **(E)** The locations of the 0 min consensus sequences (blue characters) in highest IHF binding regions, ID 56 and 57. As shown in *panel C*, position for the conserve “C” residue is also indicated with the same sky-blue arrowhead. Asterisks indicate sequences in the indicated regions.

To visualize the IHF-binding profiles and highest IHF-binding regions estimated by pmapsr on IGV, which was used for visual inspection of false IHF-binding regions (described above) and to prepare Figures (i.e., Figure 3), we independently mapped the short reads onto the reference genome using Bowtie 2 to prepare sorted BAM files (Langmead and Salzberg, 2012), the format read by IGV. Bowtie 2 was used with the default settings using Illumina short reads with the primer sequence removed by the cutadapt program (Martin, 2011). Although some of highest IHF-binding regions overlapped and were consequently included in one IHF-binding peak, we used all of highest IHF-binding peaks to estimate the IHF-binding consensus sequence in each dataset. The consensus sequences in the highest IBS were estimated using the MEME suite with default settings (Bailey et al., 2009).

### Quantitative PCR

Quantitative PCR (qPCR) experiments were performed as previously described (Kasho and Katayama, 2013; Kasho et al., 2014; Inoue et al., 2016). The levels of *oriC* and *ylcC* were quantified by real-time qPCR using SYBR Premix Ex Taq II (Perfect Real Time; Takara Bio) and primers ORI_1 and KWoriCRev for *oriC*; RTYLCC-L and RTYLCC-R for *ylcC* in SH022 or KYA018 (Figure 2B); ilvG-U and ilvG-L for ilvG, rhlB-U and rhlB-L for rhlB, glnH-U and glnH-L for glnH, osmY-U and osmY-L for osmY in KYA018 (Supplementary Figure 7); RTNoriC-U2 and RTNoriC-L2 for native *oriC*; RTTERoriC-L and RTTERoriC-L for TER-*oriC*; and RTYLCC-L and RTYLCC-R for *ylcC* in KX237 or KX238 (Figures 5B,C).

**FIGURE 4:**
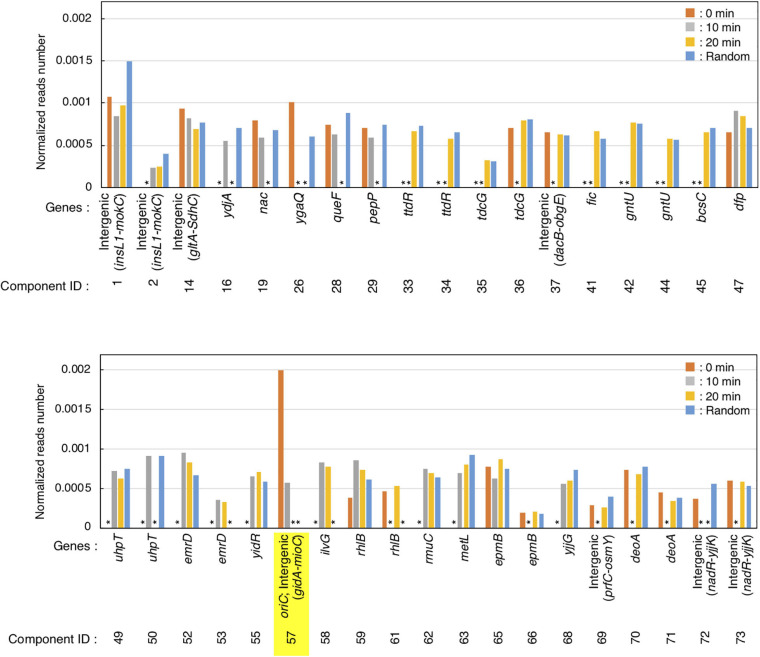
Preferential IHF binding at *oriC* at replication pre-initiation stage. Highest IHF-binding peaks at each timepoint are listed as bar charts. Y-axis indicates the normalized reads number which means the relative read numbers of each component divided by the total mapped reads numbers of the reference genome at each timepoint. Stars (*) indicate “lower than the threshold values,” which means that the component was not identified as highest IHF binding peaks at each timepoints.

**FIGURE 5:**
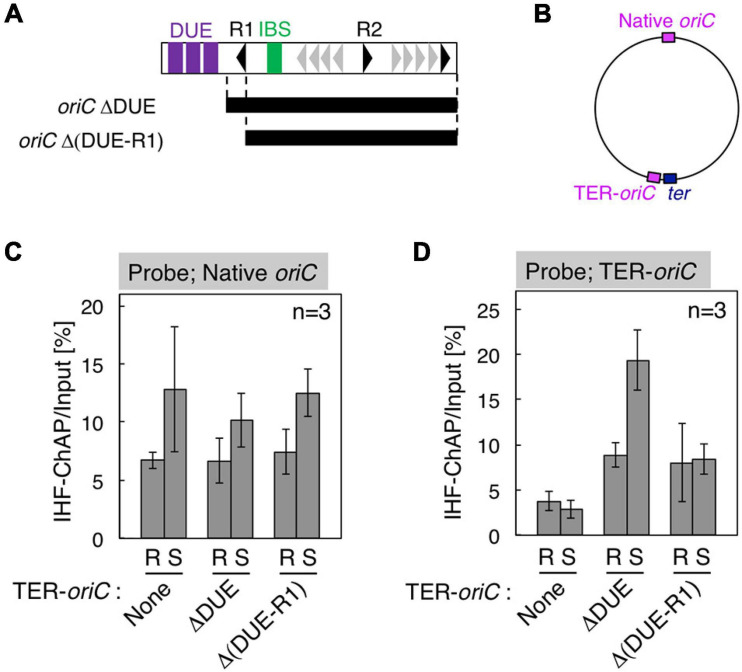
Requirement for DnaA box R1 for *oriC*–IHF binding at the replication initiation period. **(A)** Deletion derivatives of *oriC*. Bars indicate the regions of *oriC*ΔDUE and *oriC*Δ(DUE-R1), which were inserted at the position indicated as TER-*oriC* in the *terC* locus [shown in panel **(B)**] of SH022 (*dnaC2*) strain, resulting in strains KX237 and KX238. Symbols used for the *oriC* structure are shown as in Figure 1B. **(C,D)** IHF-ChAP-qPCR with TER-*oriC*-inserted cells. SH022 (*dnaC2*), KX237 (*dnaC2* TER-*oriC*ΔDUE), and KX238 [*dnaC2* TER-*oriC*Δ(DUE-R1)] cells grown at 30°C (R; Random) were transferred to 38°C and incubated for 90 min (S; Synchronized). The relative levels of native *oriC* **(C)** and TER-*oriC* **(D)** before and after Ni-affinity purification were determined using real-time qPCR, and yield was calculated (expressed as %). Error bars represent standard deviation from three independent experiments.

### Chromatin Affinity Precipitation

Chromatin affinity precipitation (ChAP) experiments were performed according to a previously described method (Kasho et al., 2014; Inoue et al., 2016).

## Results

### Specific IHF Binding to the *oriC* Locus Before Replication Initiation

Previous studies have identified or predicted thousands of IBS on the *E. coli* chromosome (Grainger et al., 2006; Prieto et al., 2012), and we recently revealed that IHF binding is dynamically regulated at two intergenic sites, *datA* and *DARS2* (Kasho and Katayama, 2013; Kasho et al., 2014), in addition to *oriC*. These observations suggest that the specific time of IHF binding is crucial for regulation of DNA replication and progression of the cell cycle. In this study, to identify the genome-wide regulation of IHF binding, we applied the GeF-seq method to temperature-sensitive *dnaC2* cells, in which it is possible to synchronize the replication cycle. DnaC is the helicase loader; in the *dnaC2* mutant, replication initiation at *oriC* is specifically inhibited at high temperatures (38–42°C) and thus we can synchronize the cell cycle just before replication initiation (0 min). By decreasing the temperature to low temperature (30°C), DnaC is immediately activated to concordantly initiate replication within 5 min in the cells. Previous studies have identified cell cycle-dependent expression of the genes such as *dnaA*, *mioC*, and *gidA* using *dnaC2* cell-based synchronization, and thus this well-established method should be the most suitable for the purpose in this study. By combining DNase I-dependent DNA cleavage, ChAP (a modified ChIP), and NGS, the GeF-seq method identifies protein-bound sites throughout the genome at base-pair resolution (Chumsakul et al., 2013).

Before mapping by GeF-seq, we confirmed by GeF-qPCR that IHF binding was regulated at the *oriC* locus in the *dnaC2* mutant, i.e., IHF stably bound to the left part of *oriC* before initiation (0 min; Figure 2) and dissociated after initiation (10 or 20 min; Figure 2), consistent with previous studies (Ryan et al., 2002; Kasho and Katayama, 2013; Kasho et al., 2014).

Next, using the same samples as in Figure 2, we performed genome-wide mapping of IHF binding at specific cell cycle stages using GeF-seq (Figure 3). Unlike standard ChIP-seq, GeF-seq includes the DNase I digestion in the DNA fragmentation process instead of sonication in ChIP-seq. As a result, protein-bound DNA fragments purified in the GeF-seq procedure are shorter than those in standard ChIP-seq and the binding peaks detected by GeF-seq become sharper than those detected by standard ChIP-seq (Chumsakul et al., 2013). In addition, we have the unique program for the GeF-seq analysis, pmapsr which was developed to accurately detect protein-binding regions on genomic DNA using GeF-seq datasets (Supplementary Table 1; Chumsakul et al., 2013). Pmapsr is the program to detect the regions sandwiched by the 5′ and 3′ ends of reads sequenced by Illumina sequencing, which are possible to represent the “edge” of the regions protected from the DNase I digestion by protein binding. Previously we succeeded to make the map of the genome wide footprinting of AbrB, the *Bacillus subtilis* global transcriptional regulator, by GeF-seq and the pmapsr analysis with similar resolution of *in vitro* DNase I footprinting and detected weak binding consensus sequence (Chumsakul et al., 2013). By this method, the presence of multiple binding regions (components) is possible to be estimated in one large peak. We, therefore, discriminate the “binding peak (peak)” and “binding region (component).”

GeF-seq results of the synchronized *dnaC2* cells showed a large peak at *oriC* in the 0 min data set, in which two possible binding regions (components) were estimated by pmapsr (Figure 3A). Those binding regions might indicate the higher-order complex formation by multiple DNA binding proteins and DNA (for instance, DnaA-IHF complexes and higher-order structures made by binding of multiple IHF molecules). In addition, our GeF-seq results included the representative IHF binding sites determined in previous *in vitro* DNase I footprinting experiments such as those at transcriptional promoters of *ilvG* or *glnH* (Supplementary Figures 1, 3; Tsui and Freundlich, 1988; Claverie-Martin and Magasanik, 1991), which supports that our GeF-seq experiments were qualified enough to determine the genomic IHF binding regions. As shown in Figure 3, the IHF-binding peak at *oriC* was prominent relative to all other IHF-binding peaks in 0 min sample (Figure 3A). To determine how specifically IHF binds to *oriC* before replication initiation, we selected highest IHF-binding peaks and binding regions, and compared the read numbers of IHF binding among those regions. Highest IHF-binding peaks (Peak ID 1-42) and binding regions (Component ID 1-73) were selected by the pmapsr program using high threshold values (see section “Materials and Methods”). Peak ID 32 (Component ID 57, genomic positions 3,925,749 to 3,925,842), highest IHF-binding peak in the 0 min dataset, overlapped with the *oriC* region (Figures 3A, 4 and Supplementary Table 1), which had the highest read depth in all of highest IHF-binding peaks selected in the 0 min dataset. The average read depth of the *oriC* locus was 30,601.5, whereas that of Peak ID 1 (Component ID 1, the genomic positions of 16,520 to 16,606), the second highest IHF-binding peak (Figure 4, Supplementary Figure 6, and Supplementary Table 1), was 16,341.3. By contrast, the same *oriC* locus in the 10 min, 20 min, and Random datasets did not have the highest read depth relative to other IHF-binding regions (Figures 3A, 4 and Supplementary Table 1), consistent with the data shown in Figure 2B and previous studies that IHF binding at *oriC* locus is increased specifically at pre-initiation period (Kasho and Katayama, 2013; Kasho et al., 2014). This observation clearly indicates that IHF binds to the *oriC* locus most preferentially before replication initiation; this was a highly distinctive property of IHF binding at the *oriC* locus, at least among highest binding regions (see next section).

In addition, before initiation, the IHF-binding peak was only prominent at the *oriC* locus, and in contrast, the signals of all other IHF-binding peaks including the *rrnC* locus, one of the rDNA operons, were relatively modest compared with the IHF binding signals at *oriC* (Figures 2B, 3A, 0 min sample). In contrast, after initiation, the IHF-binding peak at *oriC* was relatively modest or discreet, and the peaks at other loci including that at *ilvG* (Peak ID 33; Component ID 58) or the *rhlB* loci (Peak ID 34; Component ID 59 and 60) were mostly comparable or even larger compared with the IHF binding signals at *oriC* (Figure 3A, 10 and 20 min samples; Supplementary Figures 2, 3), consistent with the decreased *oriC* copies in qPCR analysis (Figure 2B). Notably, the representative IHF binding sites at transcriptional promoter of *osmY* determined in previous *in vitro* DNase I footprinting experiments was included in highest IHF binding regions (Supplementary Figure 4 and Supplementary Table 1; Colland et al., 2000), which supports that our methodology was suitable for identifying IHF binding regions. In addition, IHF binding at representative non-*oriC* loci such as *ilvG*, *rhlB*, *glnH*, and *osmY* regions was demonstrated in ChIP-qPCR experiments using IHF antibody (Supplementary Figure 7). As previously shown (Kasho and Katayama, 2013; Kasho et al., 2014), o*riC*-IHF binding was increased only at pre-initiation period (Supplementary Figure 7A). In contrast, under the same conditions, IHF binding at those non-*oriC* loci was not largely changed or even decreased in the initiation periods (Supplementary Figures 7B–E). These results are basically consistent with the GeF-seq data (see also Discussion).

### Prediction of Specific IHF-Binding Consensus Sequences Before Replication Initiation

To determine how IHF preferentially binds to *oriC* before initiation, we investigated the consensus sequences of highest IHF-binding peaks in each dataset using the MEME suite (Bailey et al., 2009). As shown in Figure 3C, the consensus sequences in all datasets (0 min: CAnnnnTTT at position 19–27, 10 min: WWTCARSnnnTTA at position 13–25, 20 min: WWCARSnnnTT at position 5–15, and Random: WAWCAACnnnTT at position 13–24, where S is G or C) are similar and contain essential DNA elements with the known consensus sequence WATCARnnnnTTR (W is A or T; n is any nucleotides; R is A or G) (Swinger and Rice, 2004). The 0 min consensus sequence is more enriched in “T/A” at positions 11–16 and 25–29 beside the conserved “CA” at position 19–20 (Figure 3C). In addition, unique “GTTG” and “AAC” elements at positions 1–4 and 31–33, respectively, locate beside these “T/A” elements (Figure 3C). The highest IHF binding region at *oriC* (Component ID 57) is one of the loci with the best match to the 0 min consensus (Supplementary Figure 5), supporting the idea that these specific DNA elements in the 0 min consensus sequence are relevant to the preferential *oriC*–IHF binding before initiation.

### Whole Genome Analysis Predicts the Preferential IHF-Binding at *oriC* Locus Before Replication Initiation

As shown in Figure 3B, we identified two IHF-protected regions (Component ID 56/57) in the prominent IHF-binding peak at *oriC* (Peak ID 32; Figure 4, Supplementary Figure 6, and Supplementary Table 1). The right protected region (Component ID 57) at *oriC* partly overlaps with a known IHF-binding consensus sequence (Figure 3E), which was previously identified as an *oriC* IBS by *in vitro* DNase I footprinting analysis (Figure 3D; Sakiyama et al., 2017). This study provides the first direct evidence for *in vivo* IHF binding to the *oriC* IBS in *E. coli* cells. The second protected region (Component ID 56) has weak IHF binding signal. Although no known IBS had been identified in this region, we identified a sequence that matches the new 0 min consensus sequence in Component ID 56 (Figures 3C,E). Alternative possibility is that the DnaA–IHF complex bound to the wide area of the *oriC* region, and the protected region would be expanded outside *oriC*. However, the existence of a secondary IBS with unstable IHF binding cannot be ruled out. Taken together, this preferential *oriC*–IHF binding before initiation requires the additional mechanism to the 0 min consensus sequences; i.e., weak IHF binding to the secondary site and the presence of two IBSs at the *oriC* locus may support stable *oriC*–IHF binding at a specific stage of the cell cycle (see below and Discussion).

We did not detect substantial specific signals at the *datA* and *DARS2* loci, although our previous study using ChIP-qPCR indicated that those should appear at 10 and 20 min after initiation. This is probably because of a difference in experimental conditions between ChIP and GeF, e.g., DNase I treatment or NGS sample preparation (see details in Discussion).

### A Role for *oriC* DnaA Box R1, but Not Chromosomal Position of *oriC*, in Stable IHF Binding

To examine the regulatory mechanism by which IHF binding at the *oriC* locus is stabilized before initiation, we focus on two specific structural features of *oriC*: (1) overall structure of 1 Mb chromosomal region containing *oriC*, termed Ori-macrodomain (Niki et al., 2000; Valens et al., 2016), and (2) the local structure of *oriC* complexed with ATP–DnaA. First, to examine the requirement of the Ori-macrodomain structure, we constructed genome-edited cells carrying an insertion of DUE-deleted *oriC* sequence at *terC*-proximal intergenic region between *pntA* and *ydgH* genes (TER-*oriC*ΔDUE; Figures 5A,B), and analyzed IHF-binding patterns before initiation. These cells have intact *oriC*s at the native position (Native *oriC*), and as expected, insertion of TER-*oriC*ΔDUE had a minimal effect on stabilization of IHF binding at the native *oriC* locus before initiation (Figure 5C). Even at the *terC* locus, the signal of IHF binding was dependent on insertion of TER-*oriC*ΔDUE, and it increased before initiation (Figure 5D). These observations suggest that timely stabilization of *oriC*–IHF binding is independent of Ori-macrodomain structure. Interestingly, IHF binding signal at TER-*oriC*ΔDUE might be higher than that at Native *oriC*, which could suggest two possibilities that DNA structure at Ter-macrodomain could be preferable for *oriC*-IHF binding, or that *oriC* DUE could play an inhibitory role for IHF binding by unidentified mechanism.

**FIGURE 6:**
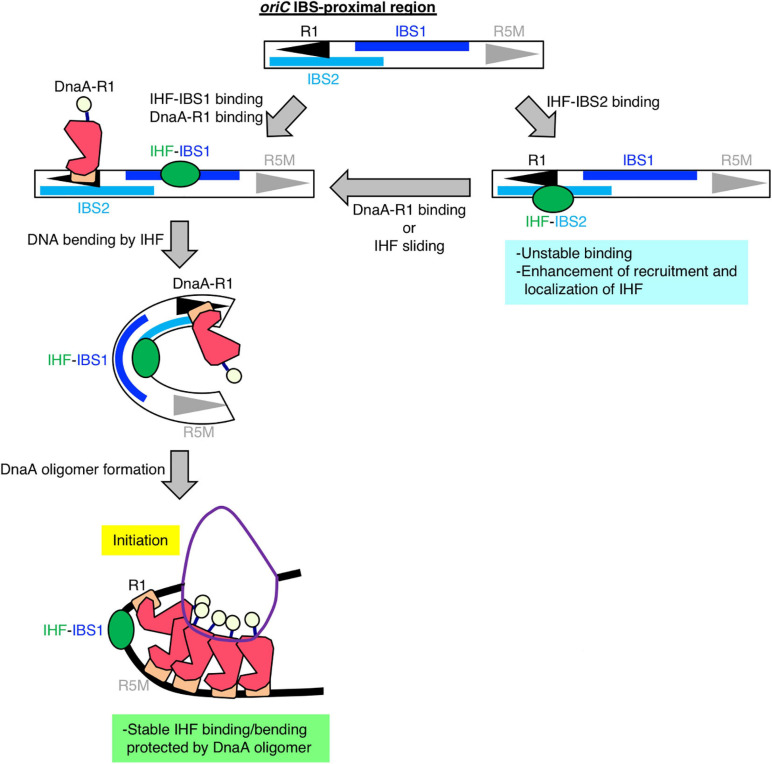
Model of DnaA box R1-mediated stabilization of IHF binding at *oriC*. Our findings suggest that, specifically at the stage of replication initiation, a secondary IBS (termed IBS2 in this study; sky-blue bar) overlaps with DnaA box R1, providing a new possible role for DnaA box R1 (black arrowhead). First, IHF binds to either a known IBS (IBS1; blue bar) or IBS2. When IHF binds to IBS2, DnaA cannot bind to R1; thus we hypothesize that even though *oriC* IBS2–IHF binding is relatively unstable, it plays a supportive role in stabilizing overall IHF binding at *oriC* by preventing free diffusion of IHF dissociated from *oriC* and promoting re-binding to *oriC*. When IHF binds to IBS1 with higher affinity and bends DNA, ATP–DnaA can form higher-order oligomers to initiate replication from *oriC*. In the initiation complex, IHF binding and bending at IBS1 is further stabilized by an ATP–DnaA oligomer, which is formed by interaction between ATP–DnaA molecules bound at R1 and another DnaA box R5 (gray arrowhead).

Second, to assess the requirement of ATP–DnaA oligomers on *oriC* for stabilizing IHF binding before initiation, we further constructed genome-edited cells carrying an insertion of an *oriC* derivative lacking a DUE-R1 region at the *TerC* locus [TER-*oriC*Δ(DUE-R1); Figure 5A]. Deletion of DnaA box R1 should drastically change the structure of ATP–DnaA oligomers on *oriC*. As with TER-*oriC*ΔDUE, insertion of TER-*oriC*Δ(DUE-R1) had little effect on the IHF-binding pattern at the native *oriC* locus (Figure 5C); at the *terC* locus, the signal of IHF binding was observed in a manner dependent on insertion of TER-*oriC*Δ(DUE-R1; Figure 5D). Notably, in contrast to the TER-*oriC*ΔDUE, the signal of IHF binding at TER-*oriC*Δ(DUE-R1) was not increased even before initiation (Figure 5D). These results indicate that DnaA box R1 is required for stabilization of IHF binding at *oriC* at the stage of replication initiation. The ATP–DnaA molecule bound at DnaA box R1 is predicted to interact with the one bound at DnaA box R5 *via* DNA bending at the primary IBS (IBS1; Figure 6) induced by IHF binding (Figures 1B,C). The ATP–DnaA oligomer formed on *oriC* might stabilize DNA bending by IHF and thus form a rigid *oriC*–IHF complex. Another possibility is that the secondary IBS (IBS2; Figure 6), which partly overlaps with DnaA box R1, might play a supporting role in stabilizing IHF binding (see details in Discussion).

### An Unique Locus Binds IHF at a Specific Time

**FIGURE 7:**
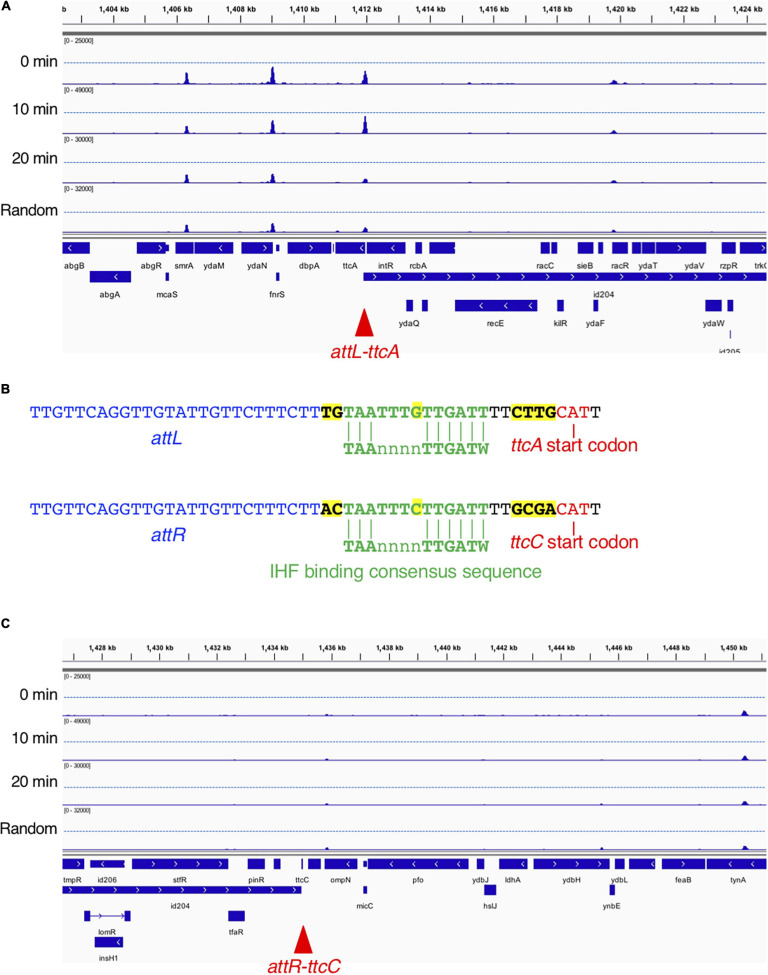
Time-resolved IHF binding at the *ttcA*-Rac prophage locus. **(A)** IHF-binding profiles at the *ttcA* upstream region in the 0 min, 10 min, 20 min, and Random samples. Red arrowhead represents the locus with IHF binding at specific timing (10 min) after replication initiation, which corresponds to Rac prophage attachment site *attL*. **(B)** Sequence comparison of Rac prophage attachment sites and IHF-binding consensus sequences in *ttcA* (top) or *ttcC* genes (bottom), and characters with yellow highlight is sequences different in *ttcA* or *ttcC* genes. The start codons of the *ttcA* or *ttcC* genes are shown by red characters. IHF-binding consensus (TAAnnnnTTGATW, where W means T or A) is shown by green characters. Rac prophage attachment sites *attL* or *attR* are shown by blue characters. **(C)** IHF-binding profiles at the *ttcC* upstream region in the 0 min, 10 min, 20 min, and Random samples. Red arrowhead represents the locus with Rac prophage attachment site *attR*.

From the genome-wide mapping of IBS by GeF-seq, we identified a novel cell cycle-dependent IHF binding in the *ttcA* gene region (Figure 7). This locus has the unique feature that IHF binds temporarily 10 min after initiation but dissociates 20 min after initiation and before initiation, as observed for all other IBSs except for *oriC* (Figure 7A). The IHF-binding peak corresponded to the IHF-binding consensus near the start codon of the *ttcA* gene, which encodes a tRNA-thioltransferase (Figure 7B; Liu et al., 2015). In addition, this locus is adjacent to the Rac prophage excision site *attL* (Figure 7B). A region including the other Rac prophage excision site *attR*, located in the *ttcC* gene, has a similar IHF-binding consensus but does not have IHF-binding peaks (Figures 7B,C). The IHF-binding region in the *ttcA* gene could play a major regulatory role in Rac prophage excision; however, this hypothesis requires further experimental testing.

## Discussion

In this study, we sought to characterize the cell cycle-dependent regulation of the genomic IHF-binding pattern using GeF-seq at base-pair resolution. The results provided evidence of unique and specific regulation of strong *oriC*–IHF binding at the replication pre-initiation stage. This suggests that at that stage, IHF preferentially binds to *oriC* rather than other sites with affinity for IHF. The mechanisms responsible for this preference remain unknown (see below). Previous studies have attempted genome-wide analysis of IHF binding by antibody-based immunoprecipitation, but no previous study detected IHF binding at the *oriC* locus (Grainger et al., 2006; Prieto et al., 2012). This is consistent with our observations that the prominent binding peak at *oriC* was specific for the initiation stage; in other stages, as well as in random cultures, the IHF-binding peaks at *oriC* were smaller than other evident binding peaks. In addition, formation of bulky *oriC*–DnaA-IHF super-complexes may inhibit the interaction between IHF and the antibodies used in previous studies. By contrast, our method, which is based on His-tag affinity purification, improves the yield of the IHF complex with bulky initiation complexes (Kasho et al., 2014; Inoue et al., 2016), and successfully detected *oriC*–IHF binding in a comprehensive analysis of genomic IHF binding for the first time.

However, our analyses also have limitations. Some of the known IHF-binding loci, such as *datA* and *DARS2*, were not substantially detected, which could be explained in three ways. First, compared with IHF binding to *oriC*, that to *datA* and *DARS2* could be more dynamic, with a rapid binding/dissociation equilibrium to ensure timely interaction during the cell cycle, making it difficult to detect the binding using the present methods. Second, in regard to cell sample preparation, our GeF-seq experiments were performed using cells at early exponential phase to determine the effect of the cell cycle on actively growing and replicating cells, whereas previous studies were performed using cells at mid/late exponential or stationary phases (Grainger et al., 2006; Prieto et al., 2012). Expression of the IHF protein is 3–6-fold higher in stationary phase than in exponential phase (Ali Azam et al., 1999), which might destabilize IHF binding to *datA* and *DARS2* in early exponential phase. Third, for the DNA samples used for NGS, protein-bound DNA was treated with DNase I to precisely determine the protein-binding site; however, previous studies suggested that DNase I may have some sequence specificities that would prevent detection of some chromosomal loci (Herrera and Chaires, 1994; Koohy et al., 2013), e.g., input read depth at the *DARS2* locus was very low relative to the surrounding regions. In addition, results obtained by ChIP-qPCR experiments were overall consistent with the GeF-seq results (Supplementary Figure 7), in certain cases relative IHF binding levels at 10 or 20 min after replication initiation were moderately different between GeF-seq and ChIP-qPCR data (Figure 3A and Supplementary Figures 7B,C). This might be caused from changes in the local copy number of genomic DNA during replication. In GeF-seq data, we cannot normalize IHF signals according to this change. Also, the IHF binding at *glnH* locus was lower than the threshold in this experiment, whereas it was detected by *in vitro* DNase I footprinting (Claverie-Martin and Magasanik, 1991). This difference might be because we set the high threshold value to identify the highest IHF binding regions which was to enable the identification of the timepoint-specific IHF binding in GeF-seq results. Therefore, the relatively lower binding peaks may also represent specific IHF binding regions. Consistently, in the previous GeF-seq experiment, many of the lower binding peaks were specific and had the consensus sequence of the *B. subtilis* transcriptional regular AbrB (Chumsakul et al., 2013). Another possible reason for the difference in the IHF binding profiles between this and previous experiments is that in experimental conditions.

Our results provide a new model for the specific regulation of local *oriC*–IHF binding at the replication initiation stage. We successfully reconstituted cell cycle-dependent IHF binding/dissociation by introducing *oriC*ΔDUE at the *terC* locus and performing *oriC* mutation analysis. The results suggested that *oriC* DnaA box R1, but not *oriC* location, is essential for stable *oriC*–IHF binding (Figure 5C). In initiation complexes, ATP–DnaA forms specific tight and bulky oligomers, which could stabilize the bending of DNA bound to IHF at the IBS-proximal region (Figure 1C), implying that ATP–DnaA oligomers might prevent IHF from freely dissociating from the *oriC* region during DNA bending promoted by IHF binding (the process is discussed below). In addition, it should be noted that in the CRISPR–Cas system of *E. coli*, IHF binding and the resultant DNA bending promotes DNA binding by the Cas1–Cas2 integrase complex, and Cas1 directly interacts with IHF (Wright et al., 2017). Similarly, IHF could directly interact with DnaA in the initiation complex. Consistent with this, HU, a structural homolog of IHF, interacts directly with DnaA (Chodavarapu et al., 2008). In addition, the possibility of a DnaA–IHF interaction could explain the appearance of an expanded protection region including DnaA box R1 (Component ID 57 in Figures 3B,E). Another possibility is that specific subcellular localization of IHF by liquid–liquid phase separation (LLPS) could occur before and at the initiation stage, followed by dramatic changes in IHF dynamics after initiation. Given that intrinsically disordered regions (IDRs) of proteins are suggested to stimulate LLPS (Borcherds et al., 2020), this hypothesis is consistent with predictions for IDRs of IHF in the MobiDB database: only α-subunits of proteobacterial IHF (such as *Salmonella typhimurium*, *Myxococcus xanthus*, *Vibrio cholerae*, etc.), but not β-subunits, have IDRs (Potenza et al., 2015). For example, amino acids 49–73 of *E. coli* IHF-α, which forms β-sheets and interacts directly with DNA (Rice et al., 1996), are predicted to be an IDR; however, further analysis is required to prove these possibilities.

Our comprehensive sequence analysis of the IHF-binding consensus suggested that before replication initiation, IHF has a specific consensus sequence that consists of conserved elements “CA” at positions 19–20 and “TT” at positions 26–27, as well as unique elements “GTTG” and “AAC” at positions 1–4 and 31–33, respectively (Figure 3C). Previous studies have tried to address the relationship between the IBS and its binding affinity; however, the requirement of the surrounding AT-rich elements in the IHF-binding consensus remains unclear (Aeling et al., 2006). This study raises the possibility that at a specific cell cycle stage, other NAPs, supercoiling state, or transcriptional profile might change the higher-order genomic structure and strengthen the requirement for surrounding AT-rich elements of the IHF-binding consensus. IHF binding to this new IHF consensus sequence might regulate expression of cell cycle-dependent genes before initiation. DnaA is known to regulate cell cycle-dependent genes such as *nrdAB* and *mioC* (Gon et al., 2006; Hansen et al., 2007). Also, SeqA protein, which specifically recognizes hemi-methylated GATC sequences after initiation, represses transcription of *dnaA* and *gidA* genes after initiation (Bogan and Helmstetter, 1997). Thus in addition to DnaA and SeqA, IHF could be a novel regulator for cell cycle-dependent gene expression.

A previous kinetic study suggested that DNA binding and bending introduced by IHF occur in a stepwise manner, and that IHF–DNA binding is a rapid process, whereas IHF-induced DNA bending is much slower and therefore rate-limiting (Sugimura and Crothers, 2006). This kinetic model and our sequence analysis of IHF-binding consensus at *oriC* suggested that *oriC* has a secondary IBS (IBS2) at a region overlapping with R1, which could also explain the mechanism by which *oriC*–IHF binding is stabilized at the replication initiation stage: first, IHF could bind to either the primary IBS (IBS1) or IBS2, and then if DnaA box R1 is occupied by DnaA, IHF can no longer bind to IBS2; in this case, however, IHF binding to IBS1 would be stabilized by *oriC*-ATP–DnaA complexes as described (Figure 6). A similar mode of discrimination of NAPs–DNA binding was proposed to occur in the regulation of *E. coli ftnA* transcription by H-NS and Fur, i.e., H-NS dimers cooperatively bind to the *ftnA* promoter to repress transcription, and Fur expression induces switching from H-NS to Fur to activate *ftnA* transcription (Nandal et al., 2010). Also, as with other DNA binding/bending proteins such as human mitochondrial transcription factor A (TFAM) (Farge et al., 2012), IHF could be mobile and slide along DNA from IBS2 to IBS1. Alternatively, IBS2 could be a reservoir of IHF, i.e., if an IHF molecule bound at the primary site IBS1 is accidentally dissociated, it could re-bind to IBS2, preventing free diffusion and thereby stabilizing overall *oriC*–IHF binding. These features of IHF-binding dynamics and IBSs in *oriC* imply that *oriC* structure is designated in a sophisticated manner to ensure IHF binding for regulation of replication initiation.

IHF dissociates from *oriC* within 5–10 min after initiation (Figures 2, 3; Kasho and Katayama, 2013; Kasho et al., 2014), although the mechanisms remain unclear. The passage of the replication machinery, which is loaded onto the unwound *oriC* region, or unknown factors could contribute to IHF dissociation. In addition, *oriC* contains eleven copies of Dam-dependent methylation sequence GATC and SeqA-dependent DnaA dissociation at post-initiation period can be considered as an analogous mechanism (Nievera et al., 2006; Katayama et al., 2010). Notably, GATC sequence is also present in IHF binding regions identified in this study (Figures 3D,E), suggesting that SeqA sequestrates DnaA and IHF from *oriC* to inhibit over-initiations. Also, after initiation, ATP–DnaA is converted to ADP–DnaA, causing ADP–DnaA to become predominant in cells (Kurokawa et al., 1999). ADP–DnaA molecules form unstable oligomers on *oriC*, suggesting that dissociation of DnaA molecules from *oriC* might impede stable interaction of IHF with *oriC in vivo*.

In addition, we identified a novel IHF-binding region at the Rac prophage excision site *attL* (Figure 7). In the CRISPR–Cas system, IHF interacts directly with Cas1 integrase and promotes the interaction of Cas1–Cas2 complexes with DNA, thereby suppressing off-target integration by Cas1–Cas2 (Wright et al., 2017). Also, IHF is a stimulatory factor for both integration and excision of bacteriophage lambda (Segall and Nash, 1996; MacWilliams et al., 1997). In *E. coli*, Rac prophage plays a role in cellular stress responses and uses a lambda phage-like mechanism for integration and excision by IntR integrase (Liu et al., 2015). Interestingly, the function of Rac prophage is stimulated under specific environmental conditions such as high nutrition or growth in early exponential phase (Liu et al., 2015), suggesting that similar significant role of IHF might be present in Rac prophage excision, and that IHF binding could stimulate the excision at Rac prophage excision site *attL* at a specific cell cycle stage, which remains to be determined. The significance of these cell cycle-specific interactions remains unknown.

## Data Availability Statement

The datasets presented in this study can be found in online repositories. Genome sequencing data were deposited in the DDBJ Sequence Read Archive (DRA) under BioProject number PRJDB11576 and accession numbers DRA012298 (0 min pulldown), DRA012299 (10 min pulldown), DRA012300 (20 min pulldown), DRA012301 (Random pulldown), DRA012302 (0 min Input), DRA012303 (10 min Input), DRA012304 (20 min Input), and DRA012305 (Random Input).

## Author Contributions

KK and TO performed the experiments. TO, OC, KN, and KF analyzed the IHF binding profile from GeF-seq data. KK, TO, and TK wrote the manuscript. All authors conceived the experiments and analyzed the data.

## Conflict of Interest

The authors declare that the research was conducted in the absence of any commercial or financial relationships that could be construed as a potential conflict of interest.

## Publisher’s Note

All claims expressed in this article are solely those of the authors and do not necessarily represent those of their affiliated organizations, or those of the publisher, the editors and the reviewers. Any product that may be evaluated in this article, or claim that may be made by its manufacturer, is not guaranteed or endorsed by the publisher.

## Footnotes

1
https://github.com/NGS-maps/gefseq01

2
https://github.com/NGS-maps/gefseq011

3
https://software.broadinstitute.org/software/igv/
