# Supplementary material for: Whole-Genome Analysis Reveals That the Nucleoid Protein IHF Predominantly Binds to the Replication Origin oriC Specifically at the Time of Initiation
Source: Front Microbiol. 2021 Aug 12;12:697712. doi: 10.3389/fmicb.2021.697712 (PMC8407004; doi:10.3389/fmicb.2021.697712)
Supplement: Supplementary file 2 [file Data_Sheet_1.PDF]

Supplementary Material

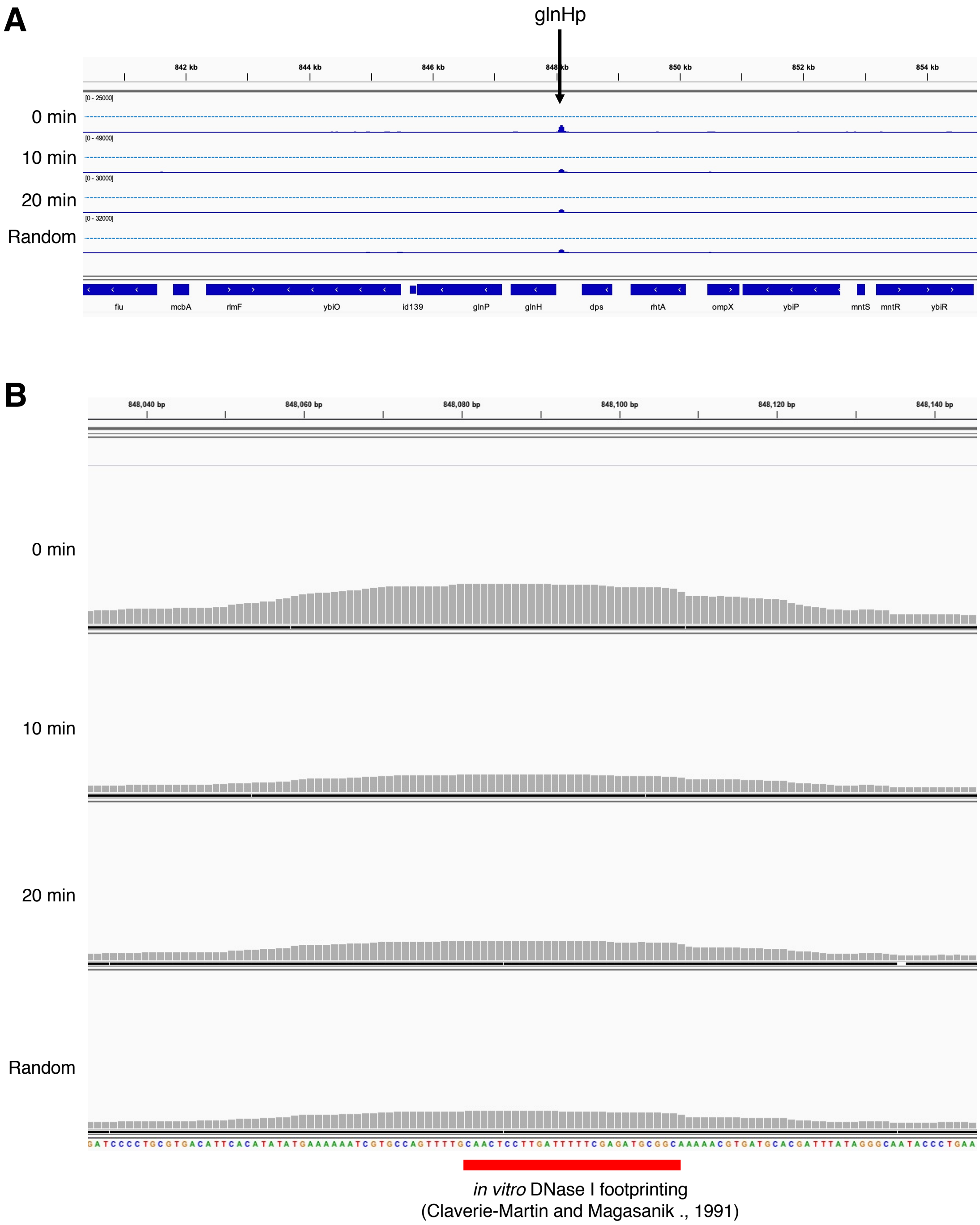

SUPPLEMENTARY FIGURE S1. IHF binding at the transcriptional promoter of *glnH* gene. (A) The IHF-binding profile (0 min, 10 min, 20 min, and Random samples) in the region including 842–854 kb part in the *E. coli* K-12 genome. (B) Extension of Supplementary Figure S2A at the *glnHp* regions. Known IBS is shown as a red bar, which was determined by *in vitro* DNase I footprinting (Claverie-Martin and Magasanik, 1991).

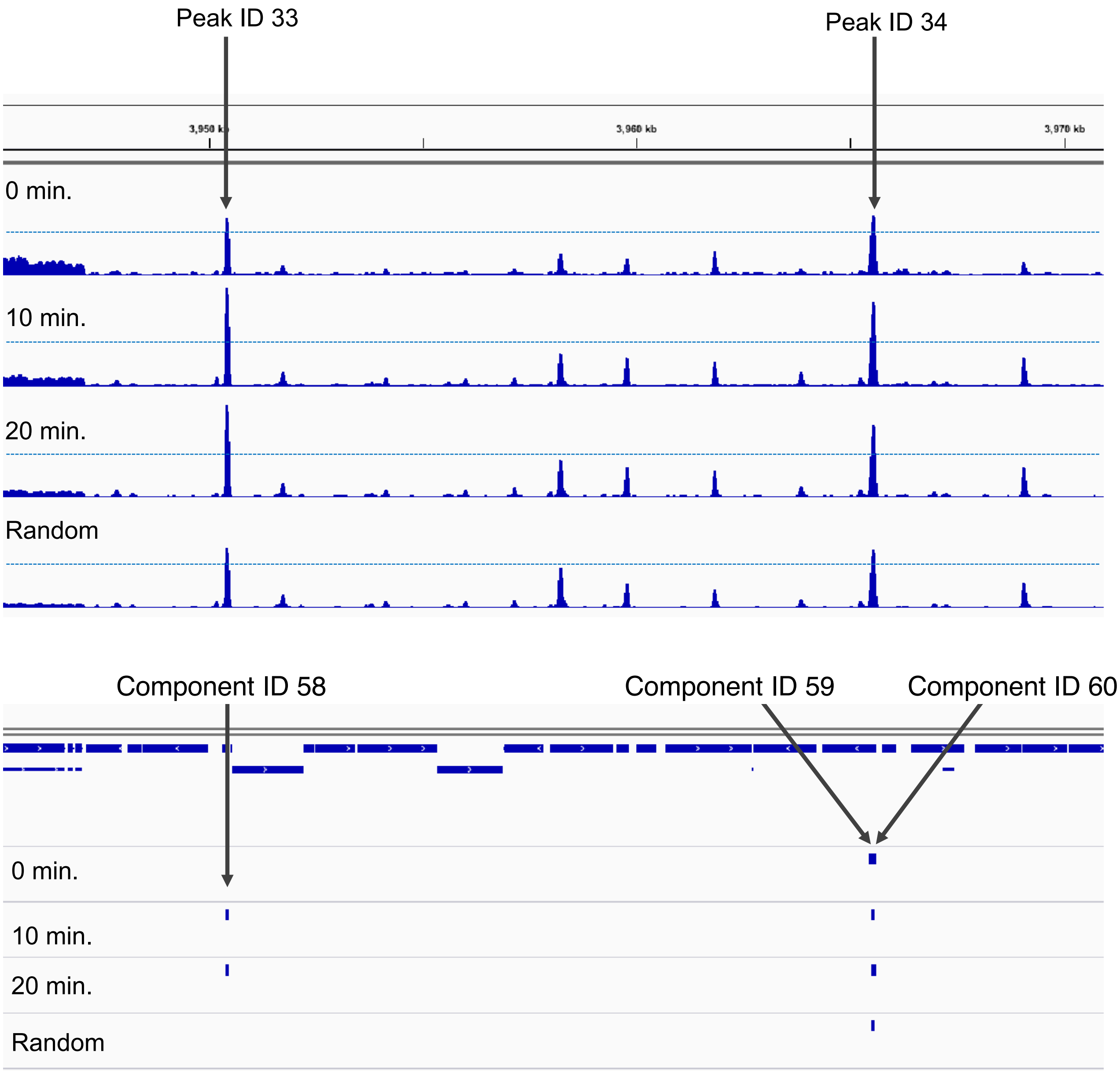

SUPPLEMENTARY FIGURE S2. Examples of IHF-binding peaks and highest IHF-binding regions. The IHF-binding profile in the region including 3950–3970 kb part in the *E. coli* K-12 genome. Upper panels indicate the locations of IHF-binding peaks in the 0 min, 10 min, 20 min, and Random samples (Peak ID 33, 34). The lower panels indicate the locations of highest IHF-binding regions included in the peaks of Peak ID 33 and 34 (Components ID 58, 59, 60). As shown in these panels, two overlapped highest IHF-binding regions were estimated in Peak ID34.

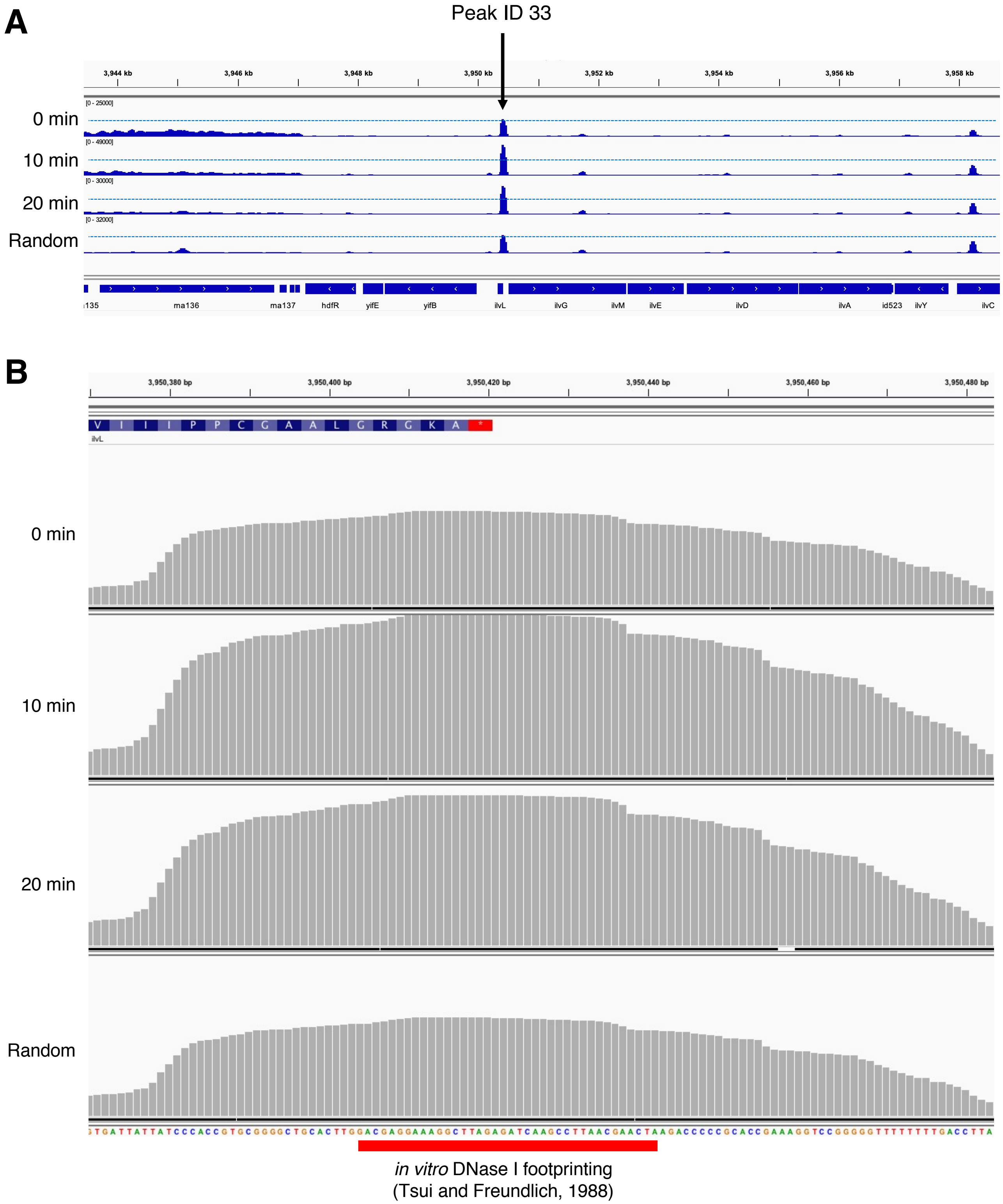

SUPPLEMENTARY FIGURE S3. IHF binding at the transcriptional promoter of *ilvG* genes. (A) The IHF-binding profile (0 min, 10 min, 20 min, and Random samples) in the region including 3944–3958 kb part in the *E. coli* K-12 genome. Threshold values are shown as blue broken lines. (B) Extension of Peak ID 33 in Supplementary Figure S1A at the *ilvGp* region. Known IBS is shown as a red bar, which was determined by *in vitro* DNase I footprinting (Tsui and Freundlich, 1988).

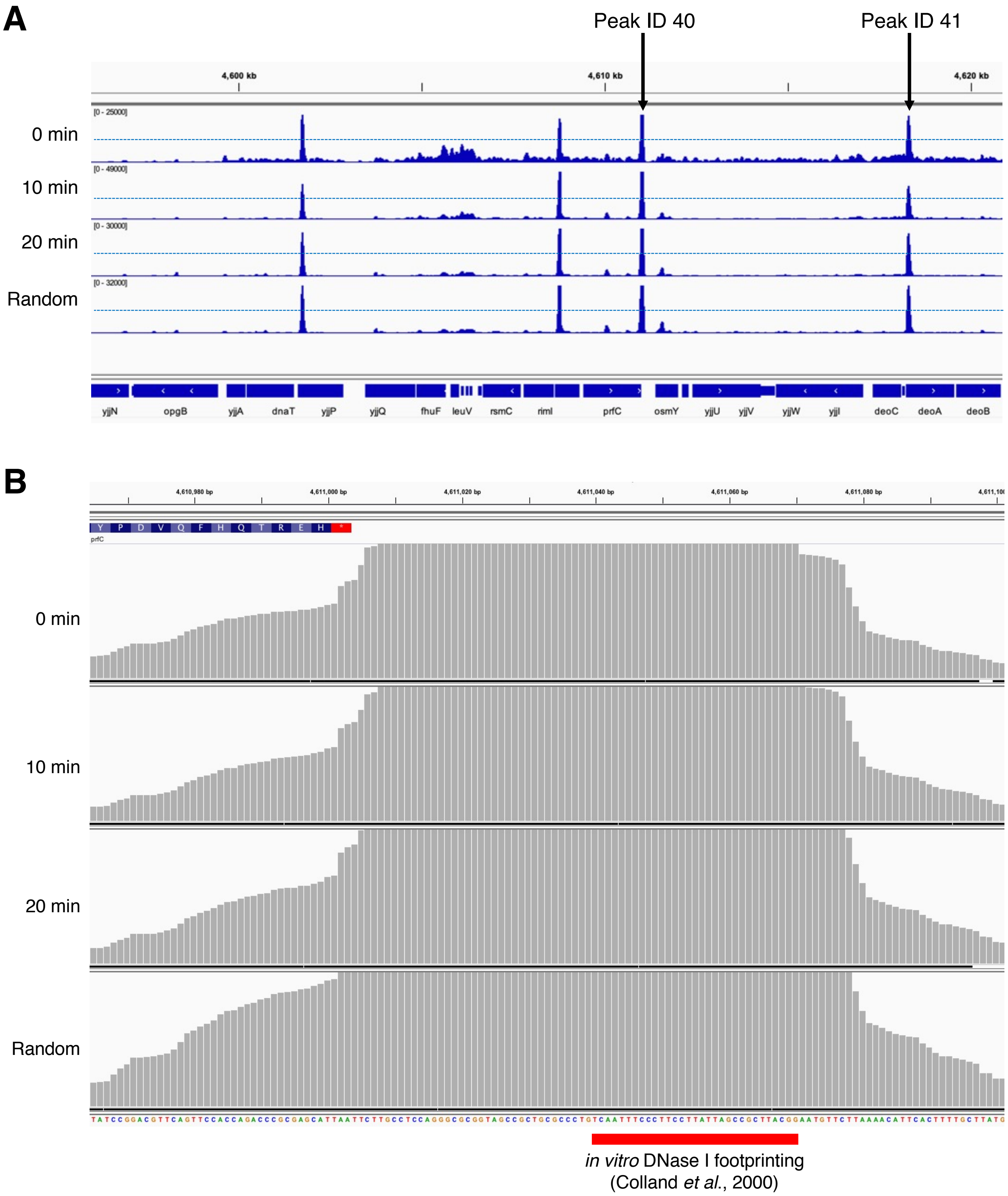

SUPPLEMENTARY FIGURE S4. IHF binding at the transcriptional promoter of *osmY* gene. (A) The IHF-binding profile (0 min, 10 min, 20 min, and Random samples) in the region including 4600–4620 kb part in the *E. coli* K-12 genome. (B) Extension of Peak ID 40 in Supplementary Figure S4A at the *osmYp* regions. Known IBS is shown as a red bar, which was determined by *in vitro* DNase I footprinting (Colland et al., 2000).

ID	Number	Positions	Strand		Match(/19)
1	16520	Intergenic	+	GTTGTGGAGAATAACAAAAATGGTCATCTGGAGCTTA	13
13	748839	ybgO	–	GTGGCGCAGTAGAAAAATCAGAAGCGATTCAACCGTT	13
19	2061766	nac	–	CTGGAAGGTGAGTTAAATCAACAACTTTTGATCCGTA	14
26	2786674	ygaQ	+	GGATAGGAGAAAGAAAAACATAGGTTTATCAACGAGA	16
29	3054464	pepP	–	CATTCAGCGAAATCAATCAGCAACTTTATCAACTACT	13
36	3259299	tdcG	+	TCTTTGCTACAGGAAAATCAACAATATGCGCACCAGA	12
47	3813768	dfp	+	GATGTTTCCCAGCCAACTCAAGGATTTAACAGCGACA	15
56	3925649	oriC	–	TTTTAATACCCAGGATCCCAGGTCTTTCTCAAGCCGA	12
57	3925749	oriC	–	GTTGTTGATCTTAAAAGCCGGATCCTTGTTATCCACA	17
60	3965491	rhlB	–	GTAGAAGCCCTTGAAAAAAAAGGGTTTCATAACTGTA	17
61	3965530	rhlB	–	GTAGAAGCCCTTGAAAAAAAAGGGTTTCATAACTGTA	17
70	4618253	deoA	+	CTGAGCGATGAAGAAAATTCGTTTCTTTATCAACGGTA	16
73	4628772	Intergenic	–	TTATCCGGCCTACAAAACCAGCAATTTCAATACGTTG	14
				GTTGnnGnnnWnnAAAnnCRnnnnTTTnWnAACnnnA	

GeF-seq 0 min. consensus sequence

SUPPLEMENTARY FIGURE S5. Determination of 0 min-specific IHF binding consensus sequence. The highest IHF binding regions used for predicting IHF binding consensus by Logos are shown. Red characters mean identical to 0 min consensus sequences as shown at the bottom. The highest IHF binding regions at *oriC* locus were labeled with yellow.

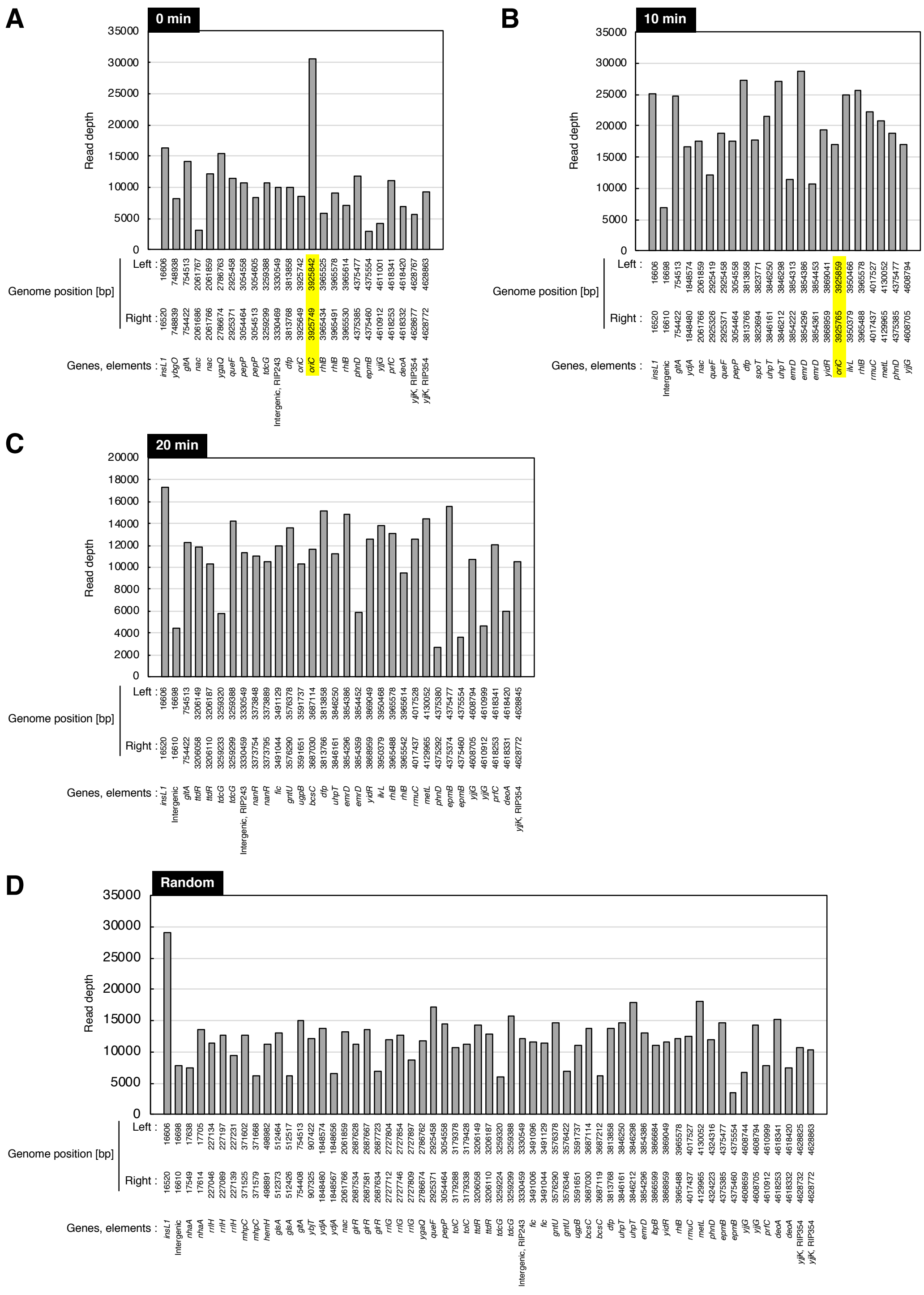

SUPPLEMENTARY FIGURE S6. Preferential IHF binding at *oriC* before initiation. Highest IHF-binding peaks were selected based on threshold values for read depths: 10000 for 0 min (A), 19606 for 10 min (B), 11697 for 20 min (C), and 12784 for Random (D). Peaks are listed as bar charts. Y-axis indicates the read depth of each IHF-binding region, and the genomic position of each region and names of genes or DNA elements overlapped with highest binding regions are shown at the bottom of each chart. The new *oriC* IBS estimated in this analysis (ID 57 in *oriC* peak: Peak ID 32) is colored in yellow.

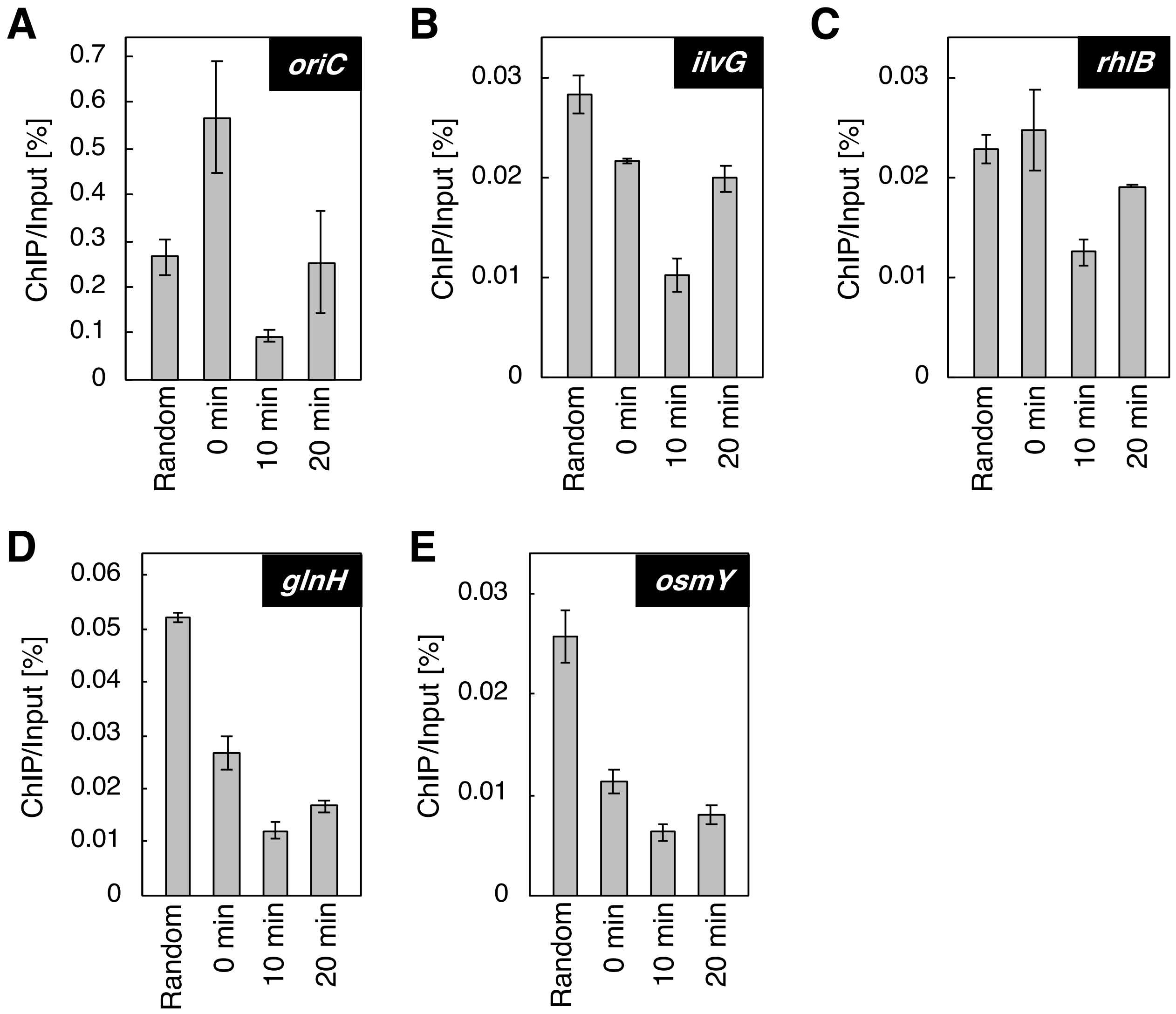

SUPPLEMENTARY FIGURE S7. Specific increase of *oriC*-IHF binding at pre-initiation period. ChIP-qPCR experiments were performed using samples prepared previously. Briefly, KYA018 [*dnaC2*] cells growing at 30 °C in supplemented M9 medium were transferred to 38°C and incubated for 90 min. The cells were then transferred to 30 °C (time 0), incubated for 10 or 20 min; samples were withdrawn at the indicated times. The *oriC*, *ilvG*, *rhlB*, *glnH*, *osmY*, and *ylcC* levels before (Input) and after (ChIP) immunoprecipitation using anti-IHF antiserum were determined using real-time quantitative PCR. The ChIP/Input for *ylcC* (%) was used as a background control for nonspecific IHF binding and was subtracted from the ChIP/ Input for *oriC*, *ilvG*, *rhlB*, *glnH*, and *osmY*. Error bars are calculated from two independent experiments.
